# Insight into the Endocrine System and the Immune System: A Review of the Inflammatory Role of Prolactin in Rheumatoid Arthritis and Psoriatic Arthritis

**DOI:** 10.3389/fimmu.2017.00720

**Published:** 2017-06-23

**Authors:** Man W. Tang, Samuel Garcia, Danielle M. Gerlag, Paul P. Tak, Kris A. Reedquist

**Affiliations:** ^1^Department of Clinical Immunology and Rheumatology, Amsterdam Rheumatology and Immunology Centre, Academic Medical Centre/University of Amsterdam, Amsterdam, Netherlands; ^2^Department of Experimental Immunology, Academic Medical Centre/University of Amsterdam, Amsterdam, Netherlands; ^3^Laboratory of Translational Immunology, Department of Rheumatology and Clinical Immunology, University Medical Center, Utrecht, Netherlands; ^4^GlaxoSmithKline, Cambridge, United Kingdom; ^5^GlaxoSmithKline, Stevenage, United Kingdom; ^6^Ghent University, Ghent, Belgium; ^7^University of Cambridge, Cambridge, United Kingdom

**Keywords:** rheumatoid arthritis, synovium, signaling and activation, clinical trials and methods, cytokines and inflammatory mediators, hormones, inflammation, macrophages

## Abstract

Rheumatoid arthritis (RA) is a chronic autoimmune disease that affects females three times more frequently than males. A potential role for hormones, such as prolactin (PRL), may in part explain this phenomenon. The risk of developing RA is increased in women who are lactating after the first pregnancy, which might be related to breastfeeding and the release of PRL. Other studies found a protective effect of PRL on RA development. Some studies have reported that hyperprolactinemia is more common in RA and serum PRL levels are correlated with several disease parameters, although others could not confirm these findings. Overall the plasma PRL levels are on average not elevated in RA. Previously, a small number of open-label clinical trials using bromocriptine, which indirectly decreases PRL levels, were performed in RA patients and showed clinical benefit, although others found the opposite effect. Locally produced PRL at the site of inflammation may have a crucial role in RA as well, as it has been shown that PRL can be produced by synovial macrophages. Locally produced PRL has both pro-inflammatory and anti-inflammatory effects in arthritis. Psoriatic arthritis (PsA) is also an autoinflammatory disease, in which the prolactin receptor is also expressed in macrophages. The aim of this review is to provide an overview of the potential role of PRL signaling in inflammatory joint diseases (RA and PsA) and its potential as a therapeutic target.

## Key Messages

– Prolactin (PRL) can be locally produced by macrophages, but also T cells and synovial fibroblasts.
– The prolactin receptor (PRLR) is expressed in synovial macrophages, lymphocytes, and fibroblasts.
– PRL interaction with its receptor can enhance or inhibit pro-inflammatory cytokine production in a cell-specific manner.
– The balance of pro- and anti-inflammatory effects of PRL, as well as potential differences in the contributions of systemic and locally produced PRL, will need to be addressed in considering PRLR antagonists as a new strategy in the treatment of inflammatory arthritis.

## Rheumatoid Arthritis

Rheumatoid arthritis (RA) is a chronic autoimmune disease, which is characterized by pain, swelling, and stiffness of the joints due to synovial inflammation that if untreated leads to progressive and irreversible destruction of cartilage and bone. The inflammatory process in the synovium is the hallmark of the disease (1). RA is one of the most common autoimmune diseases, affecting approximately 1% of the population worldwide, and females three times more frequently than males. It is associated with an increased incidence of cardiovascular morbidity and mortality (2). Due to the heterogeneity of the disease, not only seen in the clinic but also at the molecular level, it is difficult to diagnose patients based on one single diagnostic test. Therefore, RA patients have been classified for many years according to the 1987 American College of Rheumatology (ACR) criteria for RA (3). More recently, new classification criteria were developed by the ACR and the European League Against Rheumatism (EULAR), resulting in the 2010 ACR/EULAR criteria for RA (4, 5). An important difference with the 1987 ACR criteria is the use of the presence of anti-citrullinated protein antibodies, which are highly specific for RA. The presence of these autoantibodies together with IgM rheumatoid factor can be detected in the serum of 70–80% of patients and can precede the onset of clinical signs and symptoms for many years (6, 7). Their presence is associated with a more severe and destructive disease phenotype. Patients with RA can be treated with (a combination of) non-steroidal anti-inflammatory drugs, corticosteroids, disease-modifying antirheumatic drugs, and/or biological targeted therapies, such as tumor necrosis factor (TNF) inhibitors, B-cell depleting therapy, CTLA4-Ig treatment, and interleukin-6 (IL-6) receptor antibody treatment (8, 9). However, not all patients with RA respond to the available array of antirheumatic drugs. Therefore, identifying new therapeutic targets and developing effective treatment strategies remains highly important.

## Endocrine Hormones in RA

Several hormones are known to be involved or associated with inflammatory joint diseases such as RA, suggesting crosstalk between the endocrine hormones and immunity as recently highlighted (10). In addition, cortisol is known as the strongest endogenous anti-inflammatory hormone (11). RA patients have elevated cortisol levels but inadequately low in relation to ongoing inflammation, which explains the efficacy of corticosteroids in the treatment of RA.

As RA predominantly affects women, sex hormones such as prolactin (PRL) and estrogen (estradiol) have long been thought to play an important role in RA pathogenesis (12–15). Recent evidence suggests that estrogens can have both pro- and anti-inflammatory activities (16), and similar pro- and anti-inflammatory effects of PRL in RA have been recently reviewed (14, 15). For decades it has been acknowledged that in about 65% of the RA patients, disease activity diminishes during pregnancy. This may be explained by a period of transient relative hypercortisolism. A few animal studies have also suggested the beneficial effect of higher PRL levels during (the last trimester) of pregnancy in suppressing the immune responses required for successful maternal–fetal interaction (17, 18). This was also confirmed by a recent study in adjuvant-induced arthritis in which it showed hyperprolactinemia and treatment with dopamine antagonists reducing joint inflammation and pro-inflammatory cytokine production (19). Recently, it has been shown that PRL treatment and lack of PRL receptor (PRLR) signaling downregulates and upregulates, respectively, joint inflammation, osteoclastogenesis, and the expression of pro-inflammatory cytokines related to Th17 and regulatory T cells (20).

After giving birth, a reactivation of disease activity is often seen, which might be influenced by the release of PRL in the context of breastfeeding (21). The risk of developing RA is increased in women who are lactating after the first pregnancy (22, 23), although others found a protective effect on RA development (24, 25). Nowadays it is still unclear whether PRL–PRLR interaction leads to pro- or anti-inflammatory effects in RA. The discrepancies might be explained by serum PRL levels, which are measured in relatively small patients groups and targeting only (complex) systemic secreted pulsatile hormone by bromocriptine. In the last two decades, increasing evidence has shown interactions of PRL with the immune system (26–29). Homeostasis during inflammation is achieved by a balance between cytokines and endocrine hormones. Among the protein hormones, it has been most clearly documented for PRL, playing an important role in communication and regulation of the cells of the immune system (10). Here, we will summarize the known aspects of the role of PRL in RA and also proriatic arthritis (PsA).

## The Role of PRL in RA and PsA

PRL is a neuroendocrine hormone, which is mainly secreted by the anterior pituitary gland. PRL has pleiotropic functions, ranging from inducing lactation to influences on reproductive functions, calcium metabolism, and also immune reactivity (30). PRL can also be produced by cells in many extrapituitary sites (31). It has been shown that PRL can be produced by macrophages, B-cells, NK cells, T-cells, thymocytes, and peripheral blood mononuclear cells (32–35). Macrophages play a crucial role in RA as well. The relationship between PRL and RA was also suggested due to the human PRL gene, which is located on chromosome 6 close to the HLA region (36), which on its own is associated with RA. Another study has found a linkage disequilibrium between the PRL gene and major histocompatibility complex genes, known to be associated with RA (37). It has also been shown that the PRL-1149 G/T polymorphism associated significantly in Caucasian patients with RA (38).

Previous studies have shown elevated levels of PRL in RA patients compared to controls, both in females and in males (39–41), although these findings have not been confirmed in all studies (13, 42). The contradictory results can at least be partially understood by the small sample size of the studies. It is also not always clear whether fasting morning samples were obtained for the measurement of PRL. Elevated levels of PRL might be explained by enhanced systemic secretion or increased PRL production by immune cells, such as macrophages, which would suggest a correlation between PRL levels and disease activity. Studies exploring this correlative effect have produced contradicting results however. Our group has shown that in a cohort of 119 active RA patients, the percentage of patients with hyperprolactinemia was comparable to the reported incidence in the general population, and plasma PRL levels did not correlate with any of the parameters reflecting disease activity (43). Similar results were found in an independent cohort of 30 active RA patients (43). In another, independent patient group we have confirmed similar plasma PRL levels in 22 active RA patients compared to 16 healthy controls (44). In contrast, higher PRL levels have been reported in patients with psoriasis compared to control disease and healthy controls (45), and it has been suggested that PRL may play a role in the pathogenesis of psoriasis (46). Collectively, most of the evidence suggests that plasma PRL levels are on average not elevated in RA, while more validation is needed in conditions like psoriasis.

PRL levels in plasma and synovial fluid (SF) of patients with RA and osteoarthritis (OA) have been reported to be comparable, but the potential relationship between PRL levels in blood and SF has only recently been studied (47, 48). Previously, PRL was found in SF (49). We observed comparable levels of PRL in the plasma and SF of RA patients and possibly lower PRL levels in the SF compared to the plasma level in PsA. PRL is locally expressed in the synovial tissue of RA and PsA patients (50). PRL mRNA expression in the synovial tissue of RA and PsA patients positively correlates with several clinical disease parameters, including erythrocyte sedimentation rate, swollen joint count of 28 joints, visual analog scale of global disease activity, and disease activity score of 28 joints (50). This finding of locally expressed PRL in synovial tissue provides direct evidence that endocrine hormones could affect the immune system in RA and PsA. Taken together, the presence of the PRL protein in the SF and the expression of PRL in synovial tissue suggest the local production of PRL by immune cells in RA and PsA. Previously, it has been shown that lymphocytes from RA synovial tissue might synthesize PRL (51). Interestingly, monocyte-derived macrophages from RA patients can produce PRL. To have a better understanding of the type of polarization in the different diseases, and the production of PRL by those macrophages, we used monocyte-derived macrophages from healthy donors and differentiated them in RA, PsA, spondyloarthritis, and gout SF. Common macrophage agonists present in SF include CD40L, IgG complexes, TNF, and lipopolysaccharide (LPS), but we found that these stimuli failed to further modulate PRL expression in RA and PsA SF-differentiated macrophages (50). Although the component of SF responsible for stimulating macrophage PRL production remains unknown, the regulation of local PRL production by macrophages provides further evidence of the important crosstalk between the immune and endocrine systems in the inflamed compartment. Together, these data suggest that systemically secreted PRL (by the pituitary) may have a limited role in RA, except perhaps during phases of marked hyperprolactemia during breast feeding. The available data also raise the possibility that local production of PRL by immune cells (acting *via* the autocrine/paracrine loop) may be important (52, 53).

## PRL Receptor in RA and PsA

The PRLR belongs to the hematopoietin receptor superfamily and is present in atherosclerotic plaques at the sites of most prominent inflammation where it is mainly expressed by macrophages (54). Binding of PRL to its receptor activates several signaling pathways, especially the Janus kinase–signal transducer and activator of transcription pathway (55). PRL induces production of different cytokines in murine peritoneal macrophages, including interleukin-1β (IL-1β), interleukin-12β (IL-12β), interferon-γ (IFN-γ), and TNF (56). It has also been confirmed by others that PRL induces release of IL-12 in synergy with IFN-γ by murine peritoneal macrophages (57). Recently, it has been shown that PRL increases TNF expression in peripheral monocytes of RA patients (58). It was also reported that PRL, in the presence of LPS, enhanced the release of heme oxygenase-1 and vascular endothelial growth factor by human monocytes and macrophages (59). In the context of arthritis, we have shown that the PRLR is present, mainly on macrophages, but also on von Willebrand’s factor-positive endothelial cells, in the synovial tissue of RA and PsA patients (43). The expression of the PRLR is significantly higher in the synovial tissue of RA and PsA patients compared to OA, a finding confirmed independently measuring synovial mRNA expression (43).

The presence of immunomodulatory cytokines and other factors in tissue can impact on gene expression and subsequent macrophage functional responses, a process referred to as polarization; it has previously been shown that synovial tissue macrophages in inflammatory arthritis resemble polarized macrophages (60). In monocyte-derived macrophages, which were differentiated using different stimuli, we observed the highest PRLR expression in IFN-γ and IL-10-polarized macrophages. Consistent with previous literature, our data showed that PRL enhances the expression of several genes encoding for pro-inflammatory cytokines (IL-6, IL-8, IL-12β, TNF) and chemokines (i.e., CXCL3, 5, 6, and 11). Additionally, PRL enhances the production of IL-6, IL-8, and IL-12β by macrophages (43). This effect on pro-inflammatory cytokine production was also seen in RA synovial tissue explants stimulated with PRL, but not in joints and synovial fibroblasts of rodents with inflammatory arthritis, where PRL treatment and lack of PRL signaling inhibited and stimulated, respectively, pro-inflammatory cytokine production (19, 20).

## PRL(R) Antagonists as Treatment for RA and PsA?

The concept of engagement of PRL with its receptor in synovial tissue (i.e., auto-/paracrine) identifies a potential target for therapeutic intervention. This notion is supported by experimental data. In animal models bromocriptine, a dopamine agonist which indirectly reduces PRL levels, suppresses postpartum exacerbation of collagen-induced arthritis (61). Another study reported exacerbation of collagen-induced arthritis by bromocriptine (62). Also, systemically administered bromocriptine has been shown to improve signs and symptoms of RA patients (63, 64), although others could not confirm this finding (65). Similarly, a case report described amelioration of severe RA after treatment for coincidental hyperprolactinemia with cabergoline, a dopamine receptor agonist (66). Recently, it has been shown that regardless of the serum PRL levels in patients with PsA, administration of bromocriptine improves joint and skin symptoms as well (67). While these studies are interesting and hypothesis-generating, they need to be confirmed in controlled design studies.

An autocrine loop of PRL signaling may enhance the inflammatory response of activated monocytes (68). According to previous studies and data discussed above, the concept of locally produced PRL, which can activate its receptor on target cells, has emerged as a new mechanism not only in RA but also in other pathological contexts, including breast and prostate cancer (69–71). Dopamine is inappropriate for downregulating PRL production in extrapituitary tissues, therefore alternative therapeutic approaches have been developed to block PRLR-mediated signaling in stead of PRL production *per se* in target cells. It has been shown that PRL variants acting as competitive PRLR antagonists can efficiently downregulate PRLR signaling, cell survival, and/or proliferation in various breast or prostate cancer preclinical assays (70, 71). Although a phase I clinical trial with PRLR neutralizing antibodies (LFA102) in breast or prostate cancer patients showed no antitumor activity when used as monotherapy (72), the cumulative studies reviewed here support the rationale to test this approach in chronic inflammatory diseases. Further studies are needed to explore the potential of PRLR antagonists as a novel therapeutic approach in conditions like RA and PsA.

## Conclusion

The current literature is not conclusive as to whether PRL had a broad pro-inflammatory or anti-inflammatory effect in RA, rather suggesting cell-specific responses to this hormone. Systemic secreted PRL and locally produced PRL may make distinct contributions to inflammatory arthritis as systemic levels do not correlate with disease activity parameters. PRL is present in the RA SF and can be produced by macrophages and also other immune cells. The PRLR is expressed in macrophages, as well as T cells, and upon activation of the PRLR by PRL, pro-inflammatory cytokine production is induced in both macrophages and T cells. Therefore, targeting the PRLR would be an interesting novel approach in the treatment of inflammatory arthritis.

## Author Contributions

MT contributed to study conception and design, acquisition of the data, and analysis and interpretation of the data. SG contributed to study conception and design, contributed to acquisition of the data, and analysis and interpretation of the data. DG contributed to study conception and design, and analysis and interpretation of the data. PT contributed to study conception and design, and analysis and interpretation of the data. KR contributed to study conception and design, and analysis and interpretation of the data. All authors were involved in drafting the article or revising it critically for important intellectual content, and all authors approved the final version to be published.

## Conflict of Interest Statement

DG and PT are currently employees of GSK, UK. GSK had no involvement in this project. Other authors do not have competing interests.
